# Systematic analysis of ^18^F-FDG PET and metabolism, proliferation and hypoxia markers for classification of head and neck tumors

**DOI:** 10.1186/1471-2407-14-130

**Published:** 2014-02-26

**Authors:** Bianca AW Hoeben, Maud HW Starmans, Ralph TH Leijenaar, Ludwig J Dubois, Albert J van der Kogel, Johannes HAM Kaanders, Paul C Boutros, Philippe Lambin, Johan Bussink

**Affiliations:** 1 Department of Radiation Oncology, Radboud University Medical Center, P.O. Box 9101, Nijmegen 6500 HB, The Netherlands; 2 Department of Radiation Oncology (MAASTRO), GROW - School for Oncology and Developmental Biology, Maastricht University Medical Center, P.O. Box 616/23, Maastricht 6200 MD, The Netherlands; 3 Informatics and Bio-computing Platform, Ontario Institute for Cancer Research, Toronto MSG 0A3, Canada; 4 Department of Medical Biophysics, University of Toronto, Toronto, Canada; 5 Department of Pharmacology and Toxicology, University of Toronto, Toronto, Canada

**Keywords:** Head and neck cancer, Tumor characterization, ^18^F-FDG PET, Immunohistochemistry

## Abstract

**Background:**

Quantification of molecular cell processes is important for prognostication and treatment individualization of head and neck cancer (HNC). However, individual tumor comparison can show discord in upregulation similarities when analyzing multiple biological mechanisms. Elaborate tumor characterization, integrating multiple pathways reflecting intrinsic and microenvironmental properties, may be beneficial to group most uniform tumors for treatment modification schemes. The goal of this study was to systematically analyze if immunohistochemical (IHC) assessment of molecular markers, involved in treatment resistance, and ^18^F-FDG PET parameters could accurately distinguish separate HNC tumors.

**Methods:**

Several imaging parameters and texture features for ^18^F-FDG small-animal PET and immunohistochemical markers related to metabolism, hypoxia, proliferation and tumor blood perfusion were assessed within groups of BALB/c *nu*/*nu* mice xenografted with 14 human HNC models. Classification methods were used to predict tumor line based on sets of parameters.

**Results:**

We found that ^18^F-FDG PET could not differentiate between the tumor lines. On the contrary, combined IHC parameters could accurately allocate individual tumors to the correct model. From 9 analyzed IHC parameters, a cluster of 6 random parameters already classified 70.3% correctly. Combining all PET/IHC characteristics resulted in the highest tumor line classification accuracy (81.0%; cross validation 82.0%), which was just 2.2% higher (p = 5.2×10^-32^) than the performance of the IHC parameter/feature based model.

**Conclusions:**

With a select set of IHC markers representing cellular processes of metabolism, proliferation, hypoxia and perfusion, one can reliably distinguish between HNC tumor lines. Addition of ^18^F-FDG PET improves classification accuracy of IHC to a significant yet minor degree. These results may form a basis for development of tumor characterization models for treatment allocation purposes.

## Background

In the past decades, radiotherapy has become a preferred treatment modality for advanced head and neck cancer (HNC). To increase treatment outcome, radiotherapy is given in accelerated schedules and is often combined with chemotherapy and/or biologically targeted therapies [1]. HNC require a more extensive characterization than is currently performed, in order to enhance clinical prognosis estimation, to enable therapy response prediction and to give direction to tailored therapy selection from the different therapy modalities available to patients. Molecular and biological tumor characteristics, such as proliferation rate and extent of hypoxia which are known radiation-resistance mechanisms in HNC [2], can be analyzed [*e.g.* with immunohistochemistry (IHC)] next to the histopathological and anatomical tumor traits that are commonly used for therapy allocation [3]. In studies, tumors are often assessed regarding only one or a few specified biologic markers, such as hypoxia, proliferation or a certain biologic target, and based on this limited information assigned to a particular phenotype [4]. The next step to predict intrinsic tumor behavior, such as metastatic potential or probable therapy-response, would be to combine a group of biomarkers involved in multiple cellular pathways [5]. However, the optimal combination and amount of markers for various predictive assays in radiation oncology is still unknown [6]. Furthermore, even if tumors are categorized to a similar phenotype based on one characteristic, they can display discordances regarding other cellular mechanisms. For instance, equally hypoxic HNC tumors can show discrepant proliferation rates [7,8]. This may even apply for different regions within one tumor [9]. The tumor microenvironment plays an important role in the activation of cellular mechanisms [10]. Characterization of HNC, incorporating several aspects of phenotype markers representing multiple pathways influenced by intrinsic and extrinsic factors, might help pave the way for accurate distinction of individual tumors from other tumors of the same origin. A set of adequately selected parameters based on biological processes may deliver accurate all-round tumor classification for grouping of uniform tumors for treatment allocation, prediction of treatment response or distinction of patient groups with a different prognosis.

Development of such a set of parameters would best be performed in a patient cohort, taking multiple biopsies per tumor, since a single biopsy will not represent marker expression of entire tumors [11]. However, taking additional biopsies for study-purposes is often impossible to achieve. We established 14 HNC xenograft models originating from human head and neck carcinomas, with stability across several passages [12-14]. Nevertheless, biological marker expression within one tumor model displays variation after transplantation of xenograft tumors in different animals, under the influence of external and microenvironmental factors. Using these models, we can evaluate and characterize heterogeneous head and neck tumors as it were of multiple biopsies from 14 different patients. Establishment of a direction to the appropriate size of a classification parameter-set in such tumor models may be extrapolated to the clinical situation.

The availability of non-invasive functional imaging modalities broadens the range of possibilities for quantification of HNC biological traits [15,16]. Positron emission tomography (PET) with the glucose analogue 2-[^18^F] fluoro-2-deoxy-D-glucose (^18^F-FDG) is a powerful molecular imaging method exploiting increased metabolic activity of cancer cells [17]. Research is still focused on identifying the multifactorial molecular mechanisms underlying the cancer cells’ altered glucose metabolism [18]. Nonetheless, qualitative ^18^F-FDG PET is increasingly implemented before, during and after radiotherapy for HNC [19]. Quantification of differences in ^18^F-FDG tumor uptake may supplement IHC tumor characterization. In this study, we systematically analyzed an array of tumor parameters, to investigate if parameters derived from the imaging modalities ^18^F-FDG PET and IHC, singularly or in combination, could reliably distinguish different human HNC xenograft models from one another. The IHC markers were selected based on their association with ^18^F-FDG accumulation and relationship, on a molecular basis, with tumor cell metabolism, and radiotherapy-resistance mechanisms proliferation and hypoxia [20].

## Methods

### Xenograft tumor models

Ninety-eight female BALB/c *nu*/*nu* mice (Central Animal Laboratory Radboud University Medical Center) were xenografted with MEC82 (mucoepidermoid carcinoma), SCCNij or FaDu (squamous cell carcinomas) head and neck primary tumors. All lines but FaDu were derived from patient biopsies obtained in clinical studies from the Radboud University Medical Center conducted between 1996 and 2006 [21-23]. Patients gave written informed consent after approval from the Medical Ethics Committee of the Radboud University Medical Center. All research was conducted in compliance with the Helsinki Declaration and in accordance with Dutch law. Additionally, we created a xenograft model from the FaDu cell line [24,25]. SCCNij model-numbers were: 3, 59, 68, 86, 153, 154, 167, 172, 185, 196, 202 and 240. The origin of the tumor lines is described in Additional file 1: Table S1. Two-mm^3^ tumor pieces were implanted subcutaneously in the right flank in 6-8 weeks old mice. Experiments started at an average tumor diameter of 6-8 mm. Ninety-two animals were scanned per protocol; 5 mice per tumor model were used for IHC. Animals were kept in a specific-pathogen-free unit and protocols and institutional guidelines for the proper humane care and use of animals in research were followed. The Animal Welfare Committee of the Radboud University Medical Center approved all experiments.

### Small-animal PET imaging and biodistribution

Mice were fasted for 6 hours and were subsequently anesthetized using isoflurane/compressed air before ^18^F-FDG injection until the end of the experiment. Before and during scans, body temperature was kept within normal range using heated pads and heating lamps [26]. At 45 minutes before imaging, mice were injected intravenously (i.v.) through a tail vein catheter with 0.2 mL/10.2 ± 0.8 MBq ^18^F-fluoro-2-deoxyglucose (^18^F-FDG; Department of Nuclear Medicine and PET research, VU University Medical Center, Amsterdam, the Netherlands) followed by 0.1 mL saline to propel ^18^F-FDG residue from the catheter. Specific activity was > 1 GBq/μmol and radiochemical purity was always > 97% (end of synthesis). Syringes were measured in a dose calibrator before and after injection. Before imaging, bladders were largely emptied by gentle external pressure. Animals were imaged in pairs using an Inveon small-animal PET scanner (Siemens Preclinical Solutions, Knoxville, TN). Tumors were positioned in the center of the field of view. A 15-minute emission scan was acquired followed by a 400-second transmission scan, using the built-in ^57^Co source (energy window 120-125 keV) for attenuation correction. For assessment of tumor micro-environmental characteristics, mice were injected intra-peritoneally (i.p.) with 80 mg/kg hypoxia-marker pimonidazole hydrochloride (PIMO; 1-[(2-hydroxy-3-piperidinyl)propyl]-2-nitroimidazole hydrochloride; Hypoxyprobe-1, NPI Inc., Belmont, MA) at 60 minutes before sacrifice and with 50 mg/kg S-phase marker bromodeoxyuridine (BrdU; Sigma, Zwijndrecht, The Netherlands) just before imaging. The perfusion marker Hoechst 33342 (15 mg/kg, Sigma) was injected i.v. 1 minute prior to sacrificing the animals through cervical dislocation.

Tumors and normal tissues were harvested, weighed, and counted in a gamma well counter (1480 Wallac Wizard 3*”*, PerkinElmer Life Sciences, Boston, MA). The tumors were cut; one half of the tumor was immediately snap-frozen in liquid nitrogen for IHC. Radioactivity uptake in the other half of the tumor and in normal tissues was calculated as percentage of the injected dose per gram of tissue (%ID/g) (Additional file 1: Figure S1). For radioactive decay correction, injection standards were counted simultaneously.

### PET image analysis

List mode data were acquired using the default energy and coincidence timing. Data were reconstructed using 3-dimensional ordered subset expectation maximization (OSEM3D, 2 iterations) followed by maximum a posteriori (MAP, 18 iterations, β = 0.05) reconstruction optimized for uniform resolution (Siemens Inveon Acquisition Workplace, version 1.5, Siemens Preclinical Solutions) [27]. Transaxial pixel size was 0.43 mm, plane separation 0.8 mm, and the image matrix 256×256×159 [28]. PET images were analyzed using Siemens Inveon Research Workplace software. Quantification of tracer uptake in volumes of interest (VOIs) drawn around tumor and hind leg muscles on the attenuation corrected images was obtained by calculating the maximum (SUV_max_) and mean standardized uptake values (SUV_mean_). SUV was calculated as a ratio of voxel radioactivity concentration and injected activity (both decay-corrected towards start of scan) divided by body weight. SUV_mean_ for tumors was taken from a PET tumor VOI created using automatic delineation with a fixed 40% SUV_max_ threshold [29,30]. Uptake was further quantified as the ratio of mean tumor to mean muscle uptake (T/M).

### IHC staining

Frozen tumors were sectioned using a cryostat microtome. Consecutive central 5 μm thick tumor sections were mounted on poly-L-lysine coated slides and stored at -80°C until staining. Slides were scanned for vessel perfusion based on the fluorescent Hoechst 33342 signal before staining for carbonic anhydrase-9 [CA9; primary antibody (PA) biotinylated rabbit anti-CA9 (Novus Biologicals, Littleton, CO)], BrdU [PA sheep anti-BrdU (GeneTex Inc., San Antonio, TX)], PIMO [PA rabbit anti-pimonidazole (J Raleigh, University of North Carolina)], monocarboxylate transporter-4 [MCT4; PA rabbit anti-MCT4 antibody (Santa Cruz Biotechnology, Santa Cruz, CA)], glucose transporter-1 [GLUT1; PA rabbit anti-glut1 (Neomarkers Inc, Fremont, CA)], epidermal growth factor receptor [EGFR; PA goat anti-EGFR sc-03 antibody (Santa Cruz)], phosphorylated protein kinase B [pAKT; PA rabbit anti-pAKT (Santa Cruz)], and blood vessels [PA 9 F1 (rat monoclonal against mouse endothelium, Radboud University Medical Center)]. Specific staining protocols are described in the Additional file 1.

### IHC image acquisition and analysis

Tumor sections were analyzed using a digital image analysis system as described previously [31]. After scanning stained whole tissue sections, gray scale images (pixel size 2.59×2.59 μm, dynamic range 4095 grey values) were obtained and subsequently converted into binary images. Thresholds for segmentation of the fluorescent signals were interactively set above the background staining for each IHC image. Binary images were used to calculate fractions of tumor area positive for CA9, EGFR, MCT4, pAKT, GLUT1 and PIMO relative to the total viable tumor area. BrdU labeling index (LI) was determined as the number of positively stained nuclei relative to the total number of nuclei in the tumor area. Vascular density (VD; number of vascular structures per mm^2^) and perfused vessel fraction (PF) were established. These were the “IHC parameters” analyzed for combined classification accuracy. Areas of necrosis, determined using Hematoxylin and Eosin (HE) stained consecutive tumor sections, were excluded from analysis.

### Global texture analysis

IHC images were first linearly rescaled by means of their determined signal threshold, which was also used for segmentation of the fluorescent signal, in order to make image intensities comparable between IHC images of the same marker type. Global textural features comprised the mean (only for IHC), skewness and entropy (*i.e.* Shannon’s entropy, representative for global uptake heterogeneity) of the distribution of intensity values within the tumor VOI for PET and within the positively stained tumor area for IHC. In order to compute entropy, images were first discretized into equally spaced bins. For PET images we applied a bin-width of 0.5 units SUV and the bin-width for discretizing IHC images was set at 25 units. This discretization step not only reduces image noise, but also normalizes intensities across all subjects, which in turn allows for a direct comparison of entropy values between mice. Entropy was then calculated as:

$$\mathrm{entropy}=-\sum_{i=1}^{\mathrm{Nl}}P\left(i\right)\log_{2}P\left(i\right)$$

Where *P* defines the first order histogram and *P(i)* the fraction of voxels with intensity level *i. N*_*ι*_ is the number of discrete intensity levels. Developing global texture feature values was possible for most IHC markers, but not when a particular staining followed a thin ribbon-like pattern throughout tumor sections, which was the case for PF and vessels.

### Statistical methods

All analyses and plotting were performed in R statistical environment (v2.15.2) unless stated otherwise. The packages e1071 (v1.6), lattice (v0.20-13), latticeExtra (v0.6-24), hexbin (v1.26.0) and cluster (v1.14.3) were used for data processing and graphical representation.

#### Intra- versus inter-tumor line variability

A variance component analysis for all PET and IHC parameters was performed. For each parameter a linear mixed-effects model was fit with tumor line as random effect with the nlme package (v3.1-106). This produces the variance within (intra) a tumor line, the variance between (inter) tumor lines and the total (intra + inter) variance. The ratio intra/inter was calculated and used as a measure of intra-tumor line heterogeneity, as done previously [32].

#### Tumor line prediction

To assess whether (combinations of) PET and IHC parameters could distinguish tumor lines we created Random Forest models based on these parameters to predict tumor lines. A Random Forest is an ensemble classifier generated by growing a ‘forest’ of decision trees, where each tree is trained with a different bootstrap subset of tumors and parameters. Approximately a third of the samples are omitted from each tree, creating out-of-bag (OOB) data that is subsequently used to measure classifier performance [33]. For each model a Random Forest consisting of 20,000 trees (with the default number of variables randomly sampled at each split) was built using the randomForest package (v4.6-7). Performance of these predictors was assessed with the OOB error estimate and via cross validation. For cross validation the data were randomly split in training (75% of samples) and test (25% of samples) data. A Random Forest was built in the training set and evaluated in the test set as measured by the percentage of samples correctly classified. This was repeated 1000 times. Cross validation training and test sets were the same for all evaluated models, which allowed for a direct comparison between models. These analyses focused on the 72 tumors with full IHC profiling. For a few samples in this subgroup PET data (4 tumors), MCT4 data (5 tumors) and BrdU data (3 tumors) were not assessable, and median imputation was applied to fill in these missing data (e1071 package v1.6). Accuracy distributions between models were compared by a paired *t*-test.

## Results

### Small-animal ^18^F-FDG PET imaging and IHC

**Figure 1:**
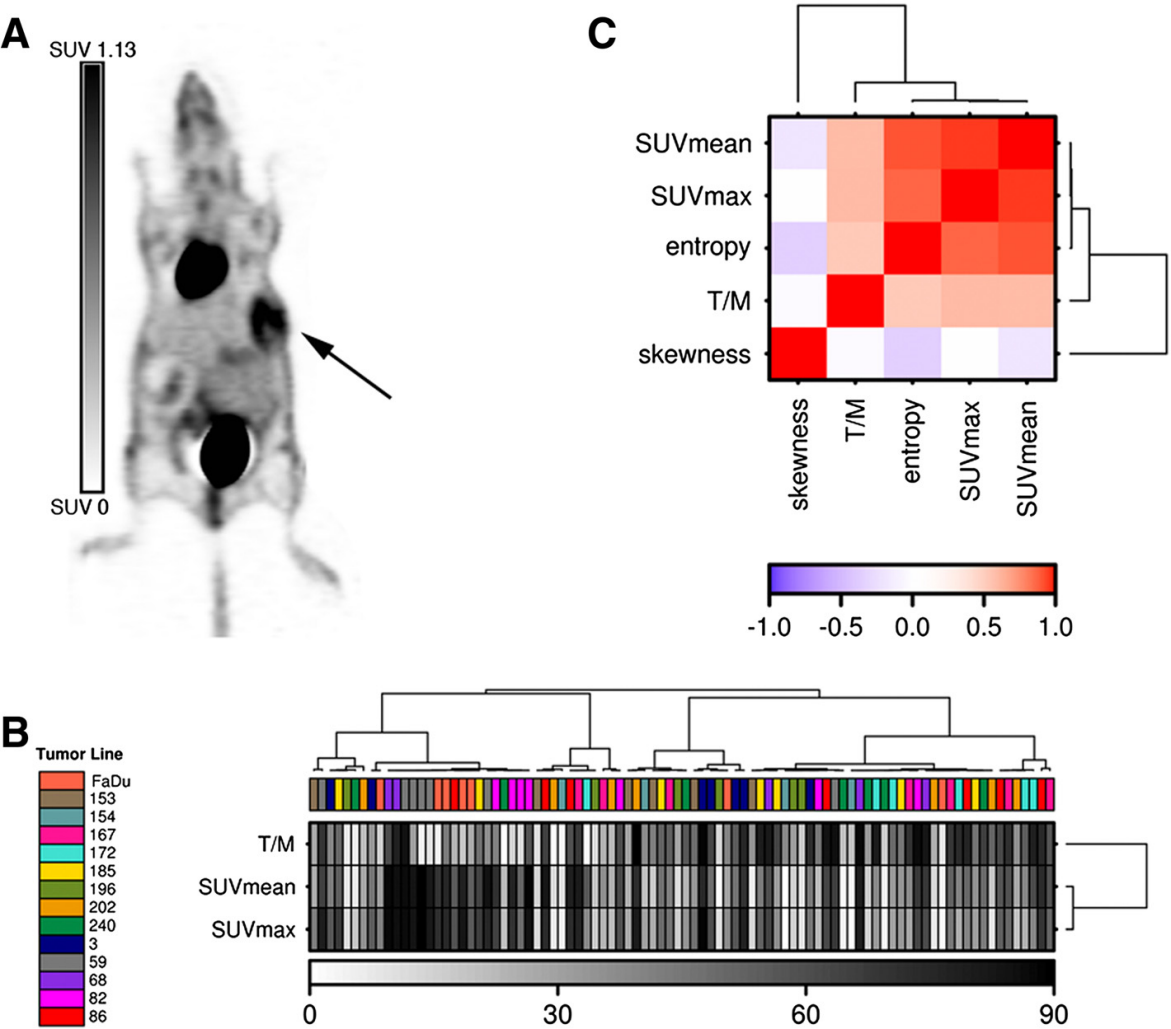
**Classification of 14 HNC lines using **^**18**^**F-FDG PET quantification parameters SUV**_**max**_**, SUV**_**mean **_**and Tumor-to-Muscle ratio (T/M) and correlation with established PET global texture features. (A) **^18^F-FDG PET image of a mouse with a head and neck xenograft tumor in the right flank (arrow). **(B)** Heatmap of the PET parameters showing no clear clustering per tumor line. Each parameter was ranked from low (white) to high (black) for analysis. Tumor lines are indicated by their respective numbers. **(C)** Correlation heatmap of the PET parameters and PET features.

For 14 different primary head and neck carcinoma xenograft models, ^18^F-FDG imaging and biodistribution was performed in 92 animals, and 72 tumors were extensively analyzed for IHC markers. An example of a PET image of a mouse with the tumor in the right flank is shown in Figure 1A. Staining parameters from IHC analysis are presented per tumor line in Additional file 1: Table S2.

#### Tumor classification using PET parameters

First, we focused on the accuracy of the PET quantification parameters SUV_max_, SUV_mean_ and T/M to allocate individual tumors to their appropriate tumor line. For each PET parameter the intra-tumor line variance was calculated as a fraction of the total variance; *e.g.* a small fraction is a measure for low intra-tumor line heterogeneity (Table 1) [32]. To assess whether PET parameters were distinct per tumor line, each parameter was ranked from low to high prior to unsupervised clustering. As Figure 1B shows, no clear clustering of the different tumor lines is observed using SUV_max_, SUV_mean_ and T/M, although SUV_max_ and SUV_mean_ are tightly correlated (Figure 1C). Next, a Random Forest was built based on the PET parameters to test their combined ability to predict the various tumor lines. The Random Forest classified samples with a 78.9% error rate, which was confirmed in cross validation: only 19.0% ± 8.0% (mean ± standard deviation SD) of samples in the test set were correctly classified (Table 2). Overall, routinely used ^18^F-FDG PET parameters were not able to distinguish a specific tumor line from the other HNC lines.

**Table 1:** Intra-tumor line heterogeneity: PET and IHC parameters and features

**Parameter**	**Texture feature**	**Within line variance**
		**Total variance**
^**18**^**F-FDG PET SUV**_**max**_		0.41
^**18**^**F-FDG PET SUV**_**mean**_		0.33
^**18**^**F-FDG PET T/M**		0.70
^**18**^**F-FDG PET**	**Entropy**	0.39
	**Skewness**	0.58
**PIMO**		0.77
	**Mean**	0.77
	**Entropy**	0.80
	**Skewness**	0.82
**BrdU**		0.78
	**Mean**	0.52
	**Entropy**	0.58
	**Skewness**	0.63
**pAKT**		0.34
	**Mean**	0.31
	**Entropy**	0.36
	**Skewness**	0.47
**EGFR**		0.29
	**Mean**	0.15
	**Entropy**	0.16
	**Skewness**	0.67
**MCT4**		0.12
	**Mean**	0.23
	**Entropy**	0.20
	**Skewness**	0.48
**CA9**		0.08
	**Mean**	0.24
	**Entropy**	0.28
	**Skewness**	0.71
**GLUT1**		0.47
	**Mean**	0.92
	**Entropy**	0.83
	**Skewness**	0.92
**Vascular density**		0.40
**Perfusion fraction**		0.55

**Table 2:** Random Forest classifier performance

	**Model**	**Accuracy overall model**	**Accuracy cross validation test set (mean ± SD)**
**PET**	**Parameters**	21.1%	19.0% ± 8.0%
	**+ features**	26.7%	23.1% ± 8.8%
**IHC**	**Parameters**	76.9%	74.9% ± 10.9%
	**+ features**	83.9%	79.8% ± 10.2%
**PET + IHC**	**Parameters**	83.6%	76.4% ± 11.0%
	**+ features**	81.0%	82.0% ± 10.6%

#### Tumor classification using PET parameters and PET features

For further analysis of the discriminatory ability of PET, global texture features derived from the individual PET images were added to the model. Although addition of the PET texture features entropy and skewness resulted in a slightly better classification accuracy (26.7%, cross validation accuracy: 23.1% ± 8.8%), these combined parameters still could not differentiate between tumor lines. The intra-tumor line heterogeneities of the PET features were in the same range as those of the PET parameters (Table 1).

#### Tumor classification using IHC parameters

**Figure 2:**
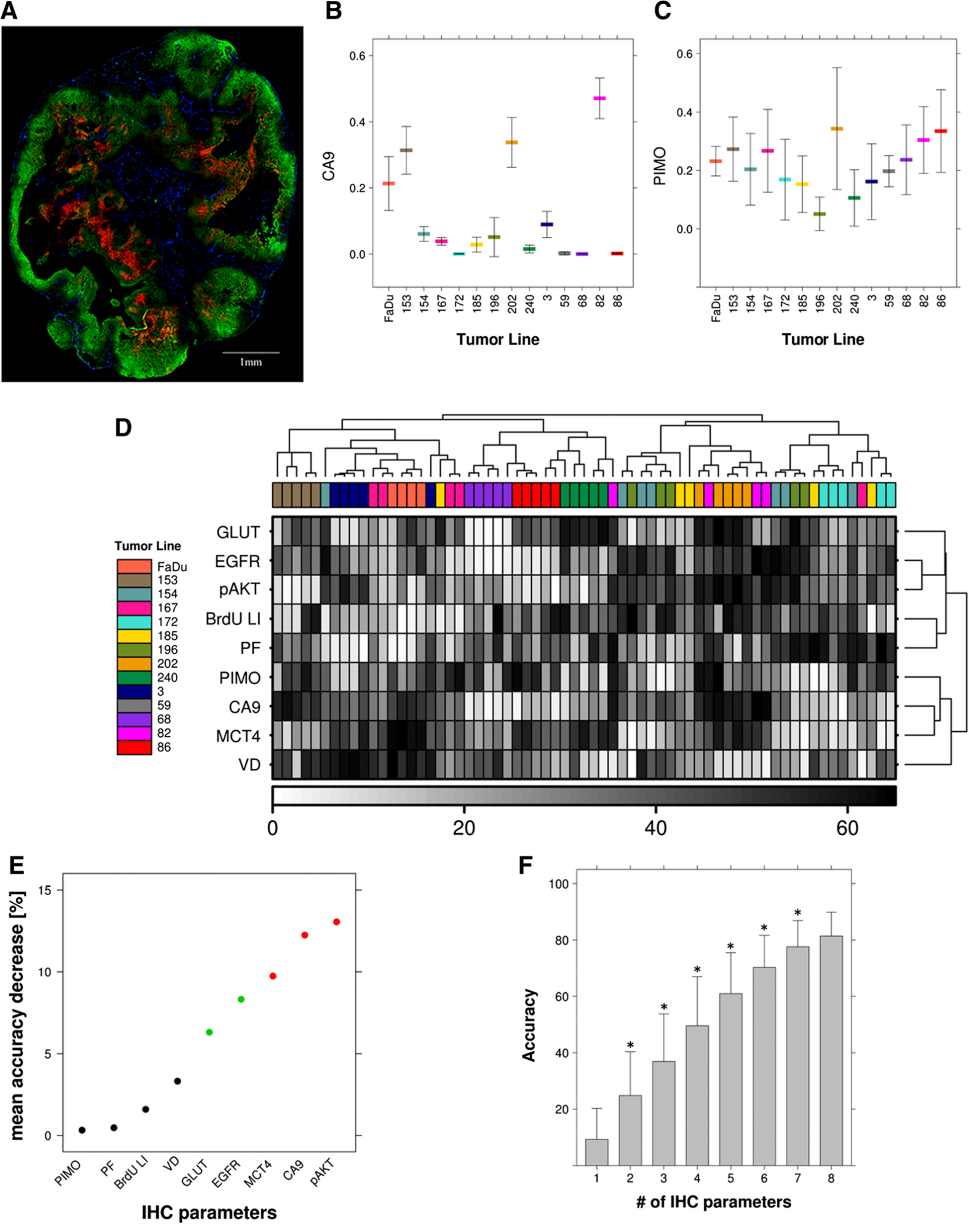
**Classification of 14 HNC lines using immunohistochemistry (IHC) marker parameters. (A)** Representative example of a combined IHC marker staining for PIMO (green), CA9 (red) and vessel (blue) staining. **(B +** **C)** Expression of an endogenous hypoxia marker (CA9) and an exogenous hypoxia marker (PIMO) in the different tumor lines (mean ± SD). **(D)** Clustered heatmap of the IHC parameters with overall good clustering of the different tumor lines. Tumor lines are indicated by their respective numbers. **(E)** Graph displaying an estimate of the decrease in Random Forest classification accuracy when omitting the respective parameter. **(F)** Random Forest classification accuracy as a function of the (randomly combined) number of IHC parameters. * = significantly different from previous number of parameters (*t*-test).

As for PET parameters, IHC staining parameters were first examined for their combined classification accuracy of the 14 tumor lines. IHC staining fractions for CA9, EGFR, MCT4, pAKT, GLUT1 and PIMO, as well as the BrdU LI, VD and PF of the microscopy-imaged tumor sections were analyzed (Figure 2A-C). Intra-tumor line heterogeneity was calculated for each IHC parameter. Notably, exogenous marker expression (PIMO, BrdU and PF) showed overall higher intra-tumor line variation than expression of the endogenous markers (Table 1, Figure 2B-C). As is shown in Figure 2D, unsupervised clustering of the IHC parameters resulted in a reasonable separation of the different tumor models.

To investigate whether the combination of IHC parameters could distinguish tumor lines, a Random Forest was built to predict the tumor line from the IHC data. Classification performance of the Random Forest was high (accuracy 76.9% as calculated from the OOB error estimate). This was confirmed in cross validation analysis, where in the test sets 74.9% ± 10.9% of the samples were classified correctly. Since each tree in Random Forest is trained on a bootstrap subset of the parameters, these can be used to estimate the importance of a parameter by calculating the decrease in classification accuracy when the parameter is omitted from a model (Figure 2E). Parameters with smaller intra-tumor line heterogeneity had a bigger effect on accuracy; these were in effect the endogenous markers. Next, we explored the influence of number of IHC parameters on classification accuracy. All possible combinations for 1 up to 8 IHC parameters were used to build a Random Forest. Classification accuracy increased significantly with the number of combined IHC parameters up to 7, and a random combination of 6 parameters already showed a classification accuracy of 70.3% ± 11.4% (Figure 2F).

#### Tumor classification using IHC features and IHC parameters

IHC global texture features were analyzed combined with IHC parameters. IHC texture features provided more information on marker distribution profiles and complemented IHC quantification values. Intra-line heterogeneity for each IHC feature is given in Table 1 and was higher for exogenous markers than endogenous markers similar to their associated IHC parameters, except for GLUT1; GLUT1 features showed greater heterogeneity than the IHC staining fraction.

**Figure 3:**
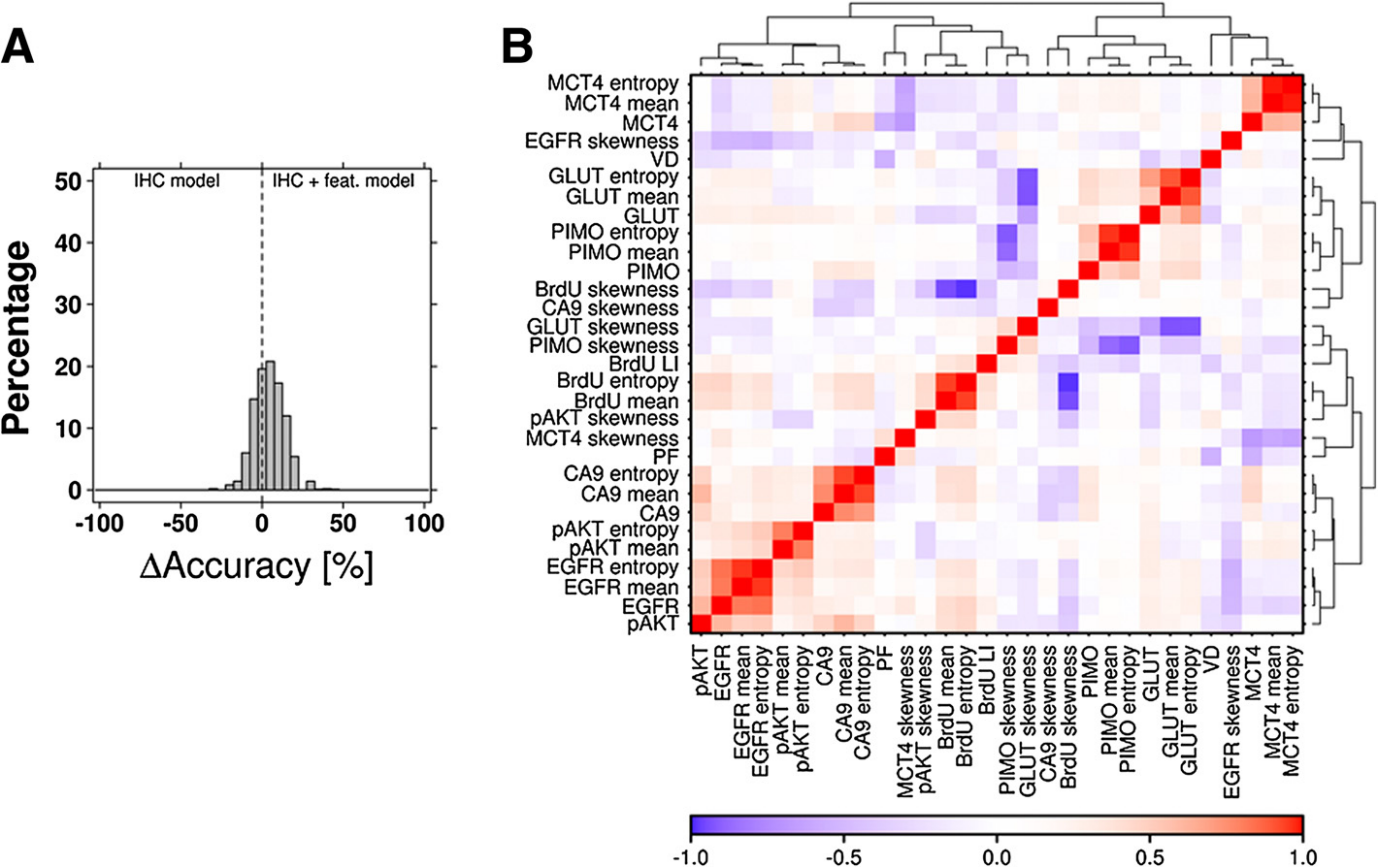
**Classification model accuracy comparison and correlation between IHC parameters and their associated texture features. (A)** Distribution of the difference in Random Forest classification accuracy of the model based on IHC parameters alone and the model based on IHC parameters combined with IHC features (feat. = features). **(B)** Correlation heatmap of the IHC parameters and the IHC features.

The addition of IHC texture features resulted in a better classification accuracy (83.9%) than using the IHC parameters alone (*i.e.* 76.9%) (Figure 3A). In cross validation 79.8% (± 10.2%) of the individual tumors were correctly classified. The Random Forest including both IHC parameters and IHC features performed significantly better than the Random Forest based on IHC parameters alone (Figure 3A, mean accuracy difference: 4.9%, 95% confidence interval [CI]: 4.2%-5.5%, p = 3.1 × 10^-47^, paired *t*-test). Furthermore, we analyzed correlations between IHC parameters and their texture features. With the exception of pAKT, the texture features mean and entropy correlated well with the associated IHC parameter (Figure 3B). The feature skewness displayed an overall negative correlation with the other IHC features and IHC parameters.

### Combination of PET and IHC parameters

**Figure 4:**
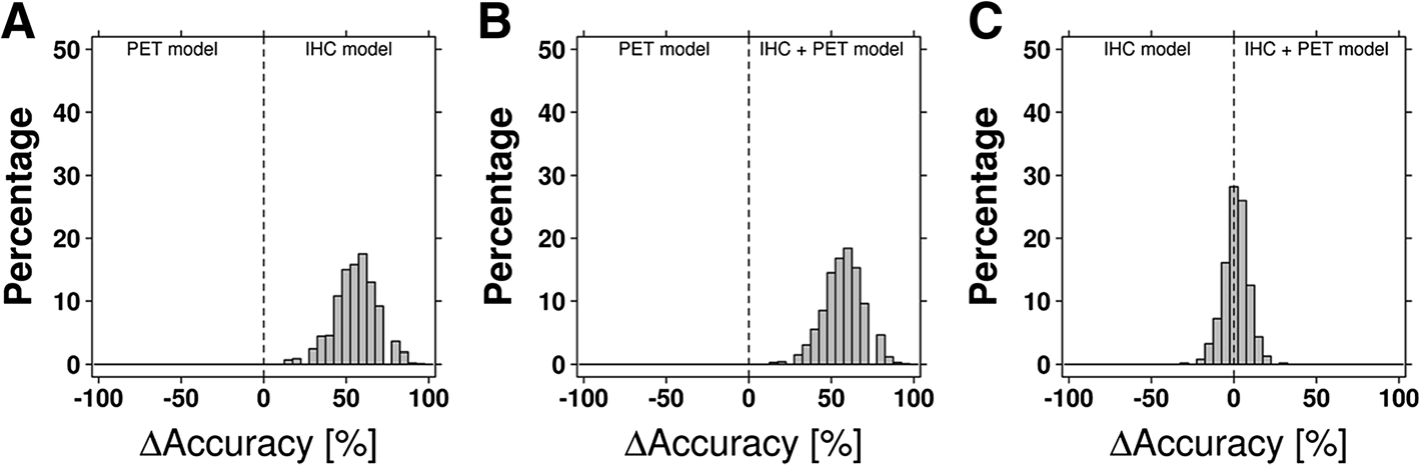
**Classification model accuracy comparison.** Distributions of the difference in Random Forest classification accuracy of models based on PET parameters versus models based on the IHC parameters **(A)**, models based on PET parameters versus models based on both PET and IHC parameters **(B)** and models based on IHC parameters versus models based on both PET and IHC parameters **(C)**.

Next, we investigated whether combining PET and IHC parameter data would result in better sample classification. Performance of the PET and IHC parameter based Random Forest was slightly better compared with the Random Forest based on IHC parameters alone (accuracy 83.6%; cross validation accuracy 76.4% ± 11.0%). The cross validation data was used to directly compare the Random Forests based on 1) PET parameters alone, 2) IHC parameters alone and 3) the combination of PET and IHC parameters with each other (Figure 4). Both the IHC based model and the combined model performed significantly better than the PET based model. Further, although the difference between the IHC based and the combined model was significant, this difference was small (mean difference: 1.4%, 95% CI: 0.9%-2.0%, p = 7.0 × 10^-8^, paired *t*-test).

### PET and IHC parameters combined with PET and IHC texture features

All texture features were added to the IHC and PET parameters to investigate whether this would further improve tumor line characterization. A Random Forest was built with these data, which resulted in an accuracy of 81.0%. In cross validation analysis, 82.0% ± 10.6% of the samples were classified correctly. The Random Forest for all data combined resulted in the highest classification accuracy.

**Figure 5:**
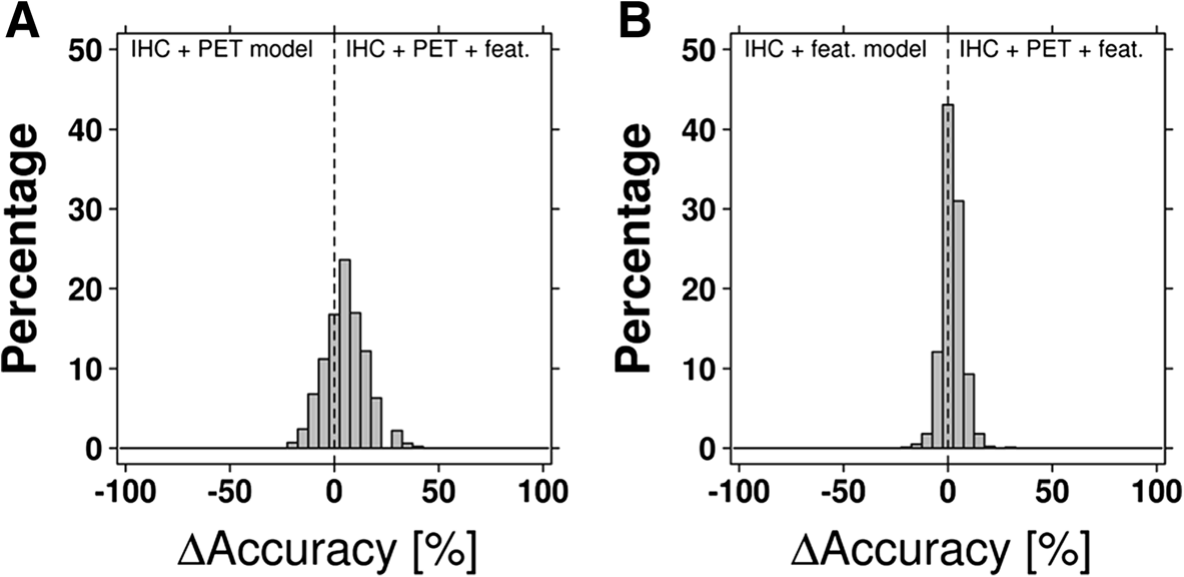
**Classification model accuracy comparison.** Distributions of the difference in Random Forest classification accuracy of models based on both IHC and PET parameters versus models based on all variables (IHC/PET parameters and features) **(A)** and models based on IHC parameters and IHC features versus models based on all variables **(B)**.

The cross validation results generated for this model were compared to data from the Random Forests based on 1) IHC and PET parameters and 2) IHC parameters and IHC features (Figure 5). Overall the model combining all parameters performed best, however differences with the IHC parameters plus IHC features based model were small (mean difference 2.2%, 95% CI: 1.8%-2.5%, p = 5.2 × 10^-32^, paired *t*-test).

## Discussion

The goal of the study was to investigate if parameters derived from ^18^F-FDG PET imaging and IHC, singularly or in combination, could reliably distinguish different human HNC xenograft models from one another. Eventually, this could give direction to classification methods for clustering of tumors that are most alike regarding multiple characteristics in clinical studies, *e.g*. for treatment prediction and prognostication purposes or for individualized treatment selection.

IHC markers were selected for relevance in metabolic cell processes and known therapy resistance mechanisms [2], as well as for (in)direct links to ^18^F-FDG tumor uptake in the literature [34-38]. Using a systematic analysis method, the presented results show that a finite set of IHC staining parameters, quantifying several relevant molecular cell processes, can accurately allocate a specific tumor to the appropriate tumor line within a cluster of 14 HNC lines. Adding more staining markers increases accuracy, but at a certain point this effect levels off. A specifying accuracy of at least 70% can be achieved with a random set of 6 of these IHC markers.

^18^F-FDG PET could not differentiate between the HNC lines in this study. Furthermore, quantification parameters (SUV, T/M) and selected ^18^F-FDG PET texture features did not provide additional value to classification accuracy by IHC alone. It may be unlikely that ^18^F-FDG PET derived parameters can reliably categorize combined differences in biological characteristics between head and neck tumors. Absolute SUVs were relatively low in this study and were in line with other preclinical HNC studies [39,40], but lower than the typical SUVs that are detected in clinical HNC [41]. This is inherent to the mouse model used for PET imaging in this study. Although differences were seen between HNC lines, most of the observed variance could be attributed to intra-tumor line differences.

Uptake of ^18^F-FDG has been assessed for correlation with several biological markers in tumors, such as GLUT1, glycolysis- and hypoxia-related markers [34,35,42], proliferation [36,42,43], EGFR [37] and AKT [38], with conflicting results. Overall, ^18^F-FDG uptake in malignancies reflects multifactorial mechanisms of increased metabolic activity and glucose utilization, performed by glucose transporters and enzymes in the glycolytic pathway, which in turn are regulated through different signaling pathways triggered by endogenous and exogenous stimulators. Aims to attribute ^18^F-FDG uptake to expression of one specific protein or therapy resistance mechanism are therefore debatable.

Quantitative texture feature analysis has been introduced in radiodiagnostic imaging as a means to characterize and classify tumors using their signal intensity distribution [44,45], and studies described the use of texture features as potential prognostic or predictive tools [46,47]. Textural feature analysis can be applied in numerous imaging modalities where lesion configuration plays a discriminating role for stratification [48], *e.g.* contact dermoscopy images [49] or microscopy images [50,51]. For this study we focused on a limited set of global features that would give relevant insight in signal distribution next to quantification parameters such as IHC staining fraction or PET SUV, including entropy and skewness for IHC and PET images, with the additional feature “mean” (pixel grey value) for IHC images. IHC texture features combined with IHC parameters conveyed optimal characterization accuracy. However, addition of 21 feature values improved the classification accuracy of the combined 9 IHC parameters (which was already 74.9%) by only 4.9%.

Limitation of the study is the use of relatively small xenograft tumors as opposed to multiple biopsies from larger HN tumors. However, this setup provides the possibility to study multiple parameters in entire tumor sections, which is difficult to achieve on a large scale in a patient setting. In clinical studies, sampling errors by extraction of a single biopsy forms a general pitfall when assessing biological markers with a heterogeneous tumor distribution. At least 4-5 central core biopsies are needed to minimize effects of IHC staining heterogeneity within tumor sections [11,52]. In entire tumors, an even greater spatial heterogeneity in IHC characteristics is likely to occur. Iakovlev *et al*. demonstrated that, for CA9 quantification in multiple cervical tumor biopsies per patient, the highest variation was inter-tumor, followed by intra-tumor and intra-tumor section variation. The greatest reduction in assessment-error could be achieved by increasing the number of biopsies spaced well apart rather than increasing the number of stained sections per biopsy [11].

We analyzed multiple tumors per xenograft model, which have the same genetic background and are grown to a similar size under similar circumstances in mice from the same strain. Even these tumors, that may represent a basic approach to multiple biopsies from heterogeneous tumors in different patients, exhibited variable characteristics during growth, affected by microenvironmental and external factors [53,54]. Intra-tumor line variation for the administered exogenous markers was overall larger than for endogenous markers. Tumor uptake of exogenous markers is influenced by dosage and administration, circulation and body clearance properties, tumor vascular density and perfusion, diffusion, binding and washout kinetics et cetera. In the clinical situation, external and microenvironmental influences may result in even larger intra-tumor and inter-tumor variation of molecular marker expression in HNC.

Results from the study can be extrapolated to other tumor types in the sense that, when the aim is to allocate or adapt individually tailored treatment, a selection of parameters provides the potential for precise tumor characterization and stratification. Depending on the treatment options at hand, individual tumor profiles or grouping of most uniform tumors can be established with the help of a distinct panel of IHC markers. This precludes analyzing an extensive number of classification parameters.

Care should be taken that the number of chosen characterizing parameters is not too small either. In this study, we found relatively low accuracies when less than 6 IHC parameters were combined for classification. Instead of administering exogenous IHC markers, molecular PET tracers with a more defined imaging spectrum than ^18^F-FDG, such as tracers for hypoxia or proliferation rate [55], can potentially complement IHC analyses by visualizing the entire tumor for presence of certain tumor mechanisms relevant for treatment.

## Conclusions

In this study, we used a systematic analysis to demonstrate that features of different quantifying methods characterize head and neck tumor lines effectively and complement each other. Multiple IHC and ^18^F-FDG PET parameters and texture features categorized individual tumors as adequate as possible. However, a select set of IHC marker parameters representing tumor metabolism, proliferation, hypoxia and blood perfusion could already allocate individual tumors to the appropriate HNC line, in an array of 14 HNC lines, with high reliability. Selected IHC texture features complemented IHC parameters for optimal characterization accuracy. ^18^F-FDG PET parameters and texture features were of minor additional value to the classification accuracy of IHC parameters alone. ^18^F-FDG as a marker may be too multifactorial influenced to distinguish microenvironmental or molecular differences between HNC lines.

## Abbreviations

BrdU: Bromodeoxyuridine; CA9: Carbonic anhydrase-9; CI: Confidence interval; 57Co: Cobalt-57; EGFR: Epidermal growth factor receptor; 18F-FDG: 2-[^18^F] fluoro-2-deoxy-D-glucose; GBq: Gigabecquerel; GLUT1: Glucose transporter-1; HE: Hematoxylin and eosin; HNC: Head and neck cancer; IHC: Immunohistochemistry; i.p.: Intraperitoneal; i.v.: Intravenous; MAP: Maximum a posteriori; MBq: Megabecquerel; MCT4: Monocarboxylate transporter-4; MEC: Mucoepidermoid carcinoma; OOB: Out-of bag; OSEM3D: 3-dimensional ordered subset expectation maximization; PA: Primary antibody; pAKT: Phosphorylated protein kinase B; PET: Positron emission tomography; PF: Perfused fraction; PIMO: Pimonidazole (1-[(2-hydroxy-3-piperidinyl)propyl]-2-nitroimidazole hydrochloride); SCC: Squamous cell carcinoma; SD: Standard deviation; SUV: Standardized uptake value; T/M: Tumor-to-muscle ratio; VD: Vascular density; VOI: Volume of interest.

## Competing interests

The authors declare no potential conflict of interest.

## Authors’ contributions

Conception and design of experiments: BH, MS, LD, AK, JK, PL, JB; Performed the experiments: BH, LD; Analyzed the data: BH, MS, RL, LD, PB; Conception and design of paper: BH, MS, RL, LD, PB, PL, JB; Wrote the paper: BH, MS, RL, JB. Revised and approved the paper: BH, MS, RL, LD, AK, JK, PB, PL, JB. All authors read and approved the final manuscript.

## Pre-publication history

The pre-publication history for this paper can be accessed here:

http://www.biomedcentral.com/1471-2407/14/130/prepub

## Supplementary Material

Additional file 1 Supplementary file. Click here for file
