# Supplementary material for: Systematic analysis of 18F-FDG PET and metabolism, proliferation and hypoxia markers for classification of head and neck tumors
Source: BMC Cancer. 2014 Feb 26;14:130. doi: 10.1186/1471-2407-14-130 (PMC3940254; doi:10.1186/1471-2407-14-130)
Supplement: Additional file 1 — Supplementary file. [file 1471-2407-14-130-S1.pdf]

Supplementary File

MATERIALS AND METHODS

Immunohistochemistry staining

Five frozen tumors per tumor line were sectioned using a cryostat microtome. Consecutive central 5 µm thick tumor sections were mounted on poly-L-lysine coated slides and stored at -80 ºC until staining. Slides were scanned for the fluorescent Hoechst 33342 signal before staining for CA9, BrdU, PIMO, MCT4, GLUT1, EGFR, pAKT, blood vessels and vessel perfusion. Between consecutive steps of the staining process, sections were rinsed 3 times for 5 min in phosphate buffered saline (PBS) (Klinipath, Duiven, The Netherlands). Primary antibodies were diluted in primary antibody diluent (PAD; GeneTex Inc., San Antonio, TX, USA), secondary antibodies in PBS.

CA9: Sections were incubated 30 min at 37 °C with biotinylated rabbit anti-CA9 (Novus Biologicals, Littleton, CO, USA) diluted 1:500. Then, sections were incubated with mouse anti-biotinCy3 (Jackson Immunoresearch Laboratories, West Grove, PA, USA) diluted 1:400 (30 min, 37 °C).

PIMO and vessels: sections were incubated with 9F1 (rat monoclonal against mouse endothelium, Radboud University Medical Center Nijmegen) undiluted (45 min, 37 °C). Next, sections were incubated with rabbit anti-pimonidazole diluted 1:1000 (30 min, 37 °C), followed by incubation with chicken anti-rat-Alexa647 diluted 1:100 (Molecular Probes, Leiden, The Netherlands) and donkey anti-rabbit-Alexa488 diluted 1:600 (Molecular Probes) for 45 min at 37 °C.

EGFR and pAKT: sections were incubated overnight at 4 °C with goat anti-EGFR sc-03 antibody (Santa Cruz Biotechnology, Santa Cruz, CA) diluted 1:50 and rabbit anti-pAKT 1:50 (Santa Cruz Biotechnology, Santa Cruz, CA, USA). This was followed by incubated with donkey anti-goat Cy3 (Jackson Immunoresearch, West Grove, PA) diluted 1:100 and donkey anti-rabbit Alexa488 diluted 1:600 for 30 min at 37 °C.

BrdU and nuclei: sections were incubated with 2N HCL for 10 min and with with 0.1 M borax for 10 min. This was followed by incubation with sheep anti-BrdU diluted (GeneTex) 1:50, overnight at 4 °C.

Next, sections were incubated with donkey anti-sheepCy3 (Jackson Immunoresearch) diluted 1:600 (30 min, 37 °C). Finally, sections were incubated with Hoechst 1 mg/mL, diluted 1:2000, at RT for 2 min.

MCT4: sections were incubated overnight at 4 °C with rabbit anti-MCT4 antibody (Santa Cruz) diluted 1:100. This was followed by incubated with goat anti-rabbit Cy3 (Jackson Immunoresearch) diluted 1:600 (30 min, 37 °C).

GLUT1: sections were incubated overnight at 4 °C with rabbit anti-glut1 (Neomarkers Inc, Fremont, CA, USA) diluted 1:100. Next sections were incubated with goat anti-rabbitCy3 diluted 1:600 (30 min, 37 °C).

After the respective staining procedures, sections were mounted in Fluorostab (ICN**,** Zoetermeer, The Netherlands).

SUPPLEMENTARY TABLES

Table S1 Xenograft origin tumor characteristics

Tumor Line	Tumor Location	TNM Classification*	Grade Diff.	HPV status	KRAS mutation status
3	Supraglottic Larynx	T2N2bM0[†]	1-2	Neg	Neg
59	Hypopharynx	T4N2cM0[‡]	2	Neg	Neg
68	Hypopharynx	T2N2cM0[‡]	2	Neg	Neg
82*	Cheek / Parotid*	T3N0M0[‡]	3	Neg	Neg
86	Supraglottic Larynx	T2N2bM0[‡]	3	Neg	Neg
153	Supraglottic Larynx	T3N2cM0[§]	2	Neg	Neg
154	Hypopharynx	T2N2cM0[§]	2	Neg	Neg
167	Supraglottic Larynx	T4N2cM0[§]	3	Neg	Neg
172	Supraglottic Larynx	T4N0M0[§]	2	Neg	Neg
185	Supraglottic Larynx	T4N1M0[§]	2	Neg	Neg
196	Hypopharynx	T4N0M0[§]	1	Neg	Neg
202	Supraglottic Larynx	T4N0M0[§]	2	Neg	Neg
240	Transglottic Larynx	T3N2cM0[§]	1	Neg	Neg
FaDu	Hypopharynx	T≥2N≥2c	2	Neg	Neg

Parotid = Parotid Gland; HPV = Human Papilloma Virus.
All tumors are squamous cell carcinomas (SCCNij and FaDu), except * = mucoepidermoid carcinoma (MEC). HPV and KRAS mutation status were analysed in frozen xenograft material [HPV: SPF LiPA method [1]; KRAS mutation analysis as described by Knijn *et al.* [2]]. Staging acccording to the [†]4th, [‡]5th and [§]6th Edition of the UICC TNM Classification

1. Kleter B, van Doorn LJ, Schrauwen L, Molijn A, Sastrowijoto S, ter Schegget J, Lindeman J, ter Harmsel B, Burger M, Quint W: **Development and clinical evaluation of a highly sensitive PCR-reverse hybridization line probe assay for detection and identification of anogenital human papillomavirus**. *J Clin Microbiol* 1999, **37**:2508-2517.
2. Knijn N, Mekenkamp LJ, Klomp M, Vink-Borger ME, Tol J, Teerenstra S, Meijer JW, Tebar M, Riemersma S, van Krieken JH*, et al*: **KRAS mutation analysis: a comparison between primary tumours and matched liver metastases in 305 colorectal cancer patients**. *Br J Cancer* 2011, **104**:1020-1026.

Table S2 ^{18}F-FDG PET, biodistribution and IHC parameters

Tumor Model	SUV_{max}	$SUV_{mean40\%}$	T/M	ID/g%	EGFR	pAKT	PIMO	CA9	MCT4	GLUT1	BrdU LI	PF
3	1.4 ±0.3	0.8 ±0.1	3.7 ±0.9	5.3 ±1.1	0.1 ±0.06	0.06 ±0.05	0.2 ±0.1	0.09 ±0.04	0.4 ±0.07	0.1 ±0.1	0.09 ±0.01	0.4 ±0.2
59	1.8 ±0.3	1.1 ±0.2	3.3 ±1.3	8.3 ±5.8	0.0005 ±0.0003	0.005 ±0.004	0.2 ±0.05	0.002 ±0.005	NA	0.2 ±0.1	0.1 ±0.1	0.8 ±0.08
68	1.6 ±0.2	1.0 ±0.1	4.3 ±0.7	6.7 ±2.1	0.003 ±0.003	0.004 ±0.003	0.2 ±0.1	0.0003 ±0.0004	0.2 ±0.04	0.03 ±0.01	0.2 ±0.2	0.7 ±0.1
82	1.4 ±0.2	0.9 ±0.2	3.1 ±1.0	6.1 ±1.5	0.4 ±0.2	0.1 ±0.06	0.3 ±0.1	0.5 ±0.06	0.4 ±0.07	0.3 ±0.1	0.2 ±0.04	0.7 ±0.1
86	1.4 ±0.2	0.9 ±0.1	3.7 ±0.8	6.3 ±1.3	0.01 ±0.01	0.001 ±0.001	0.3 ±0.1	0.0002 ±0.0002	0.4 ±0.05	0.3 ±0.03	0.2 ±0.2	0.7 ±0.07
153	1.1 ±0.1	0.6 ±0.1	2.2 ±1.1	5.1 ±1.5	0.2 ±0.08	0.0006 ±0.0004	0.3 ±0.1	0.3 ±0.07	0.2 ±0.04	0.3 ±0.1	0.2 ±0.2	0.7 ±0.06
154	0.8 ±0.3	0.5 ±0.1	2.3 ±0.6	3.1 ±0.6	0.4 ±0.06	0.06 ±0.02	0.2 ±0.1	0.06 ±0.02	0.1 ±0.07	0.3 ±0.08	0.2 ±0.1	0.7 ±0.2
167	1.2 ±0.2	0.8 ±0.1	3.6 ±0.5	5.8 ±1.3	0.02 ±0.01	0.003 ±0.001	0.3 ±0.1	0.04 ±0.01	0.3 ±0.09	0.2 ±0.09	0.08 ±0.03	0.6 ±0.2
172	1.0 ±0.4	0.7 ±0.3	3.4 ±1.2	5.0 ±0.5	0.2 ±0.1	0.007 ±0.003	0.2 ±0.1	0.0007 ±0.0007	0.07 ±0.04	0.2 ±0.07	0.2 ±0.1	0.8 ±0.08
185	1.2 ±0.2	0.8 ±0.1	3.3 ±0.5	5.2 ±0.4	0.09 ±0.06	0.03 ±0.02	0.2 ±0.1	0.03 ±0.02	0.2 ±0.09	0.3 ±0.1	0.09 ±0.05	0.7 ±0.1
196	0.8 ±0.2	0.5 ±0.1	2.1 ±0.4	3.1 ±1.7	0.4 ±0.07	0.08 ±0.08	0.05 ±0.06	0.05 ±0.06	0.08 ±0.07	0.2 ±0.2	0.4 ±0.3	0.7 ±0.1
202	1.1 ±0.3	0.7 ±0.2	2.7 ±1.5	4.6 ±1.1	0.3 ±0.03	0.2 ±0.07	0.3 ±0.2	0.3 ±0.08	0.5 ±0.09	0.5 ±0.05	0.3 ±0.2	0.7 ±0.2
240	1.0 ±0.2	0.6 ±0.1	2.2 ±0.9	4.5 ±1.3	0.1 ±0.1	0.003 ±0.002	0.1 ±0.1	0.02 ±0.01	0.4 ±0.07	0.5 ±0.03	0.2 ±0.2	0.7 ±0.1
FaDu	1.3 ±0.3	0.8 ±0.2	2.2 ±0.5	6.0 ±1.1	0.1 ±0.1	0.003 ±0.003	0.2 ±0.05	0.2 ±0.08	0.7 ±0.05	0.3 ±0.1	0.05 ±0.02	0.3 ±0.2
**All tumors**	1.2 ±0.3	0.8 ±0.2	3.0 ±1.0	5.3 ±2.2	0.2 ±0.2	0.04 ±0.06	0.2 ±0.1	0.1 ±0.2	0.3 ±0.2	0.3 ±0.1	0.2 ±0.2	0.7 ±0.2

Data are presented as Mean ± SD. SUV=Standardized Uptake Value; EGFR / pAKT / PIMO / CA9 / MCT4 / GLUT1=fraction of marker staining in viable tumor area of tumor section; BrdU LI = BrdU labeling index; PF = fraction of perfusion in tumor vessels; NA = not assessable

SUPPLEMENTARY FIGURE

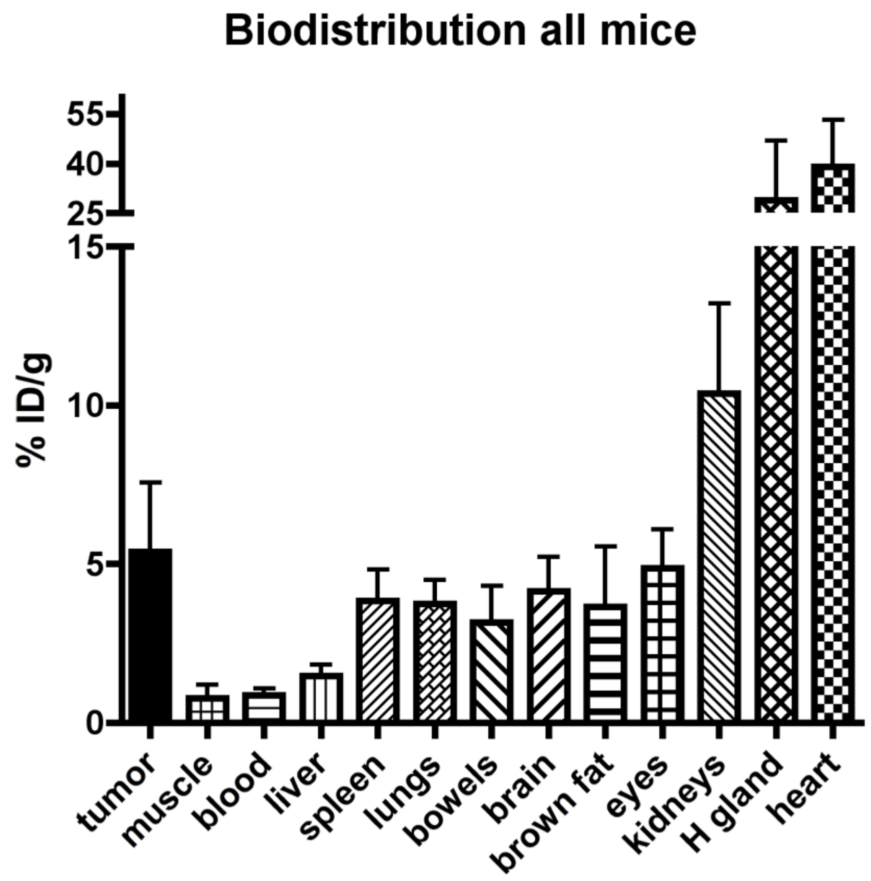

Supplementary Figure S1. Biodistribution mean uptake ± SD data of all resected tumors and normal tissues as measured by gamma well counter. % ID/g = percentage of the injected dose per gram of tissue. H gland = Harderian gland.
